# Anti-Thermal-Quenching Phosphors Based on Metal Halides

**DOI:** 10.1021/acsenergylett.5c03473

**Published:** 2025-12-22

**Authors:** Baowei Zhang, Liberato Manna

**Affiliations:** † College of Chemistry, 12636 Zhengzhou University, Kexue road, Zhengzhou 450001 China; ‡ Nanochemistry, 121451 Istituto Italiano di Tecnologia, Via Morego 30, 16163 Genova, Italy

## Abstract

Thermal quenching (TQ) generally occurs in phosphors and is ascribed to the activation of nonradiative transitions at elevated temperatures. This effect limits the use of most phosphors in high-power/high-temperature applications, such as outdoor lighting and laser systems. To achieve anti-TQ properties, structural design of phosphors is required. This usually follows two guidelines: (1) increasing lattice rigidity to minimize thermal expansion; (2) converting thermal energy into radiative transitions to compensate for the nonradiative losses. While metal oxides and metal nitrides dominate the field of commercial anti-TQ phosphors, metal halides, despite their inherently soft lattices, have shown remarkable progress as anti-TQ phosphors in recent years. Here, we review the advances in anti-TQ metal halides (covering the time span from 2017, when the first reports appeared, until today) and discuss their mechanisms and applications. We argue that the low synthesis temperatures of metal halides and their high photoluminescence quantum yields (PLQYs) make them promising candidates as anti-TQ phosphors. Furthermore, since the rich optical–physical processes underlying the anti-TQ effect in soft-lattice in metal halides are only now beginning to be unraveled, this creates opportunities for many fundamental investigations.

## Graphical abstract

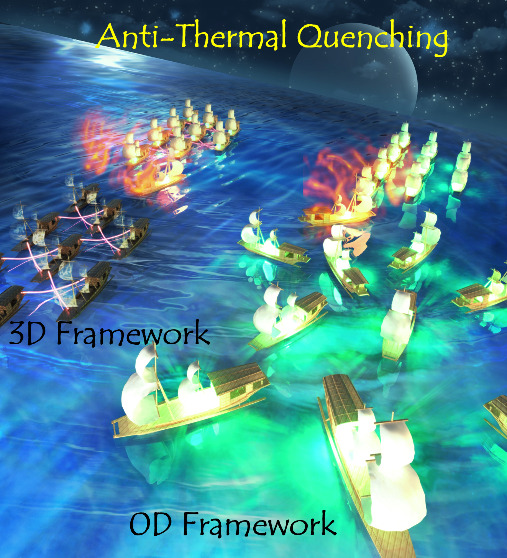

High luminous power and brightness is essential for outdoor lighting that significantly extends human productive/social time.^1^ Yet, in most phosphors high-power operation raises device temperatures up to 200 °C,^2^ triggering thermal quenching (TQ) of the emission and leading to energy waste. To limit the losses in the performance and efficiency of high-power lighting devices, two strategies are employed: (1) external thermal management by enhancing heat dissipation with the use of heat sinks or high-thermal-conductivity media, to limit increases in device temperature under high power operation conditions;^3^ (2) intrinsic phosphor design, that is, deployment of anti-TQ phosphors as emitters.^4^ This second strategy will be the focus of this review, which will highlight recent developments on anti-TQ phosphors based on metal halides.

Fundamental studies on anti-TQ phosphors date back to the 1960s, when G. Blasse established the theoretical framework for metal oxide phosphors doped with lanthanide (Ln) ions.^5–7^ In the top three diagrams of Figure 1, the blue upward arrow represents the absorption process, while the green downward arrow denotes the emission process. TQ occurs when thermal excitation (red zigzag line) promotes the system from the excited-state minimum (B) to the ground/excited-state crossover point (C). As sketched in Figure 1a,d, complete TQ occurs at any given temperature when C is the global minimum of excited states. As shown in Figure 1b,e, TQ is possible even without the assistance of thermal energy (*E* = *kT*, where *k* is the Boltzmann constant and *T* is the environment temperature), when C lies above the minimum point of the excited state curve (point B), but below the energy level reached after absorption (point A). As shown in Figure 1c,f, anti-TQ occurs when C lies above A, that is, when the thermal energy is not enough to surmount the energy barrier (Δ*E*).

**Figure 1:**
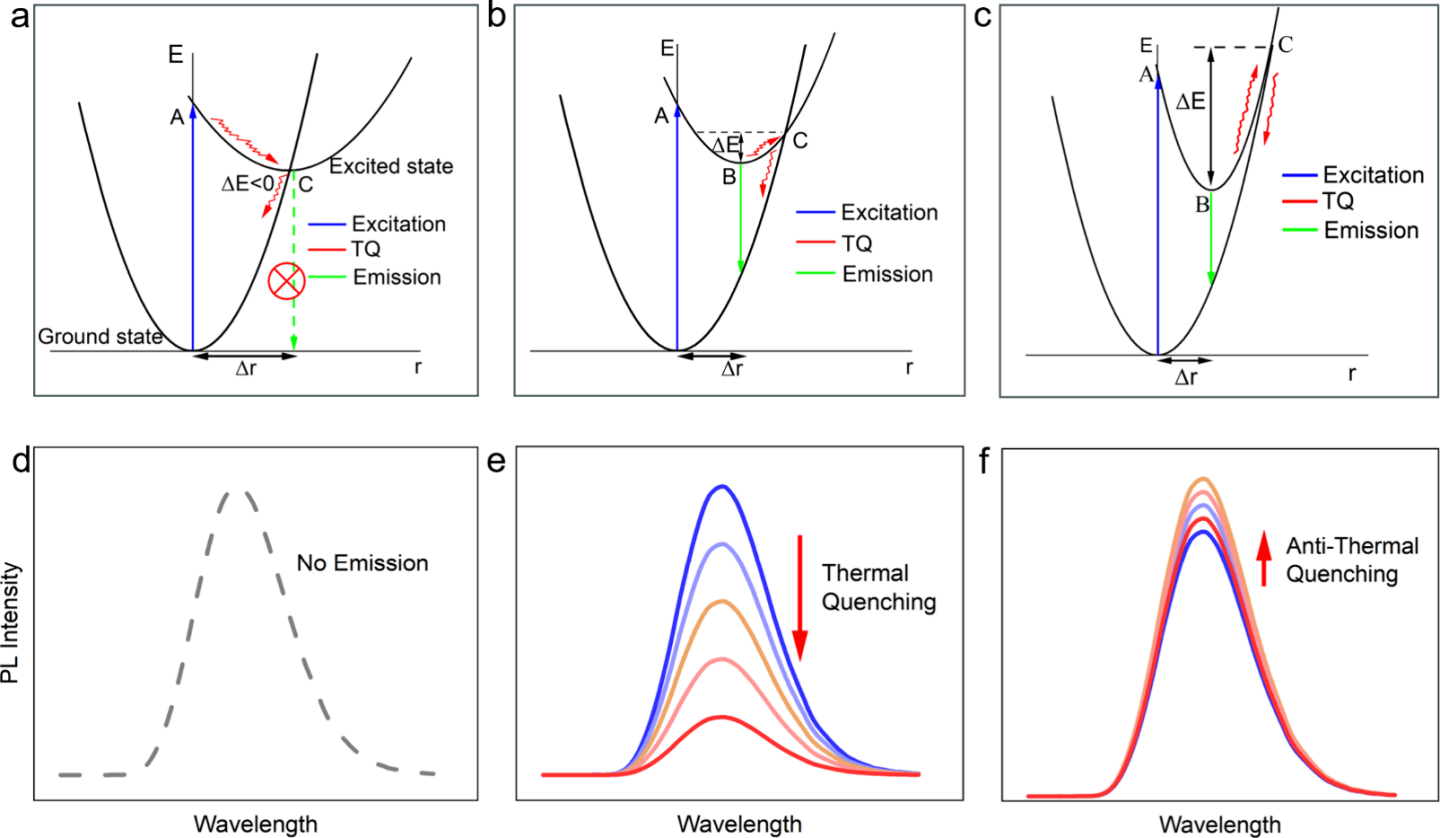
Three different configuration coordinate diagrams (a–c) for thermal quenching processes and their corresponding thermal quenching behavior (d–f), based on the model proposed by G. Blasse in 1974.^7^ Reproduced (Adapted) with permission from ref 7. Copyright 1974, Elsevier. In panels a–c, *r* is the configuration coordinate representing the average position of the atoms surrounding a luminescent center, while Δ*r* represents the difference between the equilibrium distance of the ground and excited state.

Mainstream anti-TQ systems are based on metal oxides and metal nitrides.^8,9^ The underlying feature of these materials is their rigid structure characterized by a small thermal-lattice expansion coefficient.^2,5,10^ For example, Kim et al. reported in 2017 a blue-emitting Na_3–2*x*_Sc_2_(PO_4_)_3_:*x*Eu^2+^ phosphor with zero-thermal quenching emission at 200 °C,^2^ and Qiao et al. developed in 2018 a K_2_BaCa­(PO_4_)_2_:Eu^2+^ phosphor that preserved 100% of its room temperature PL intensity at 275 °C.^11^ Metal halides, on the other hand, represent a broad class of materials characterized by [M^*m*+^X_*n*_]^*m*−*n*^ polyhedral skeletons (M = metal, X = Cl, Br, I) that are often charge-stabilized by A^+^ cations (A^+^ = K^+^, Rb^+^, Cs^+^, or an organic cation).^12^ Metal halides are generally not considered as anti-TQ phosphors, as they tend to have “soft lattices”.^13^ Nevertheless, they have gained increasing attention in recent years (Table 1), following the discovery by Yuan et al. in 2017 of anti-TQ behavior from Mn^2+^ doped CsPbCl_3_ relatively to the emission from Mn^2+^ states.^14^ Anti-TQ metal halides fall into three categories: (1) metal halides doped with metal ions except Ln ones (for example Mn^2+^, Sb^3+^, Mo^4+^), in which the thermally assisted energy transfer from host/defects to the emission center can compensate the energy losses of the emission center, so that the phosphor can reach stable emission even at high temperature;^4^ (2) metal halides doped with Ln ions, in which the 4f–4f transitions of the Ln elements are insensitive to the coordination environment, so they preserve their emission characteristics under thermal lattice expansion;^15,16^ (3) metal halides that have undergone a surface treatment (so far documented only for lead halide perovskite nanocrystals) to form a wide bandgap surface layer, allegedly leading to a type-I core–shell structure that can mitigate the TQ process.^17,18^ Research on anti-TQ phosphors based on metal halides is a relatively new field of research, born only eight years ago and now experiencing a boost. This field is not yet covered by any review so far, hence the rationale for this focus review.^19^

**Table 1:** Composition, Synthesis Temperature (°C), Emission Peak (nm), PLQY (%), and *T*_c_ (K) for Metal Halide and Metal Oxide/Nitride Anti-TQ Phosphors Covering the Emission Spectrum from the Whole Visible Range to the Near Infrared (NIR)^a^

Material class	Composition	Synthesis temperature (°C)	Emission peak (nm)	PLQY (%)	*T*_c_ (K)
Metal halides	^20^CsCdCl_3_:*x*%Br^–^	180	482	84	377
	^4^Rb_3_InCl_6_:*x*%Sb^3+^	110	521	90	500
	^21^Rb_3_(Cd_0.8_Mn_0.2_)_2_Cl_7_	150	575	88	423
	^22^Cs_2_ZnCl_4_:*x*%Sb^3+^	150	745	75	500
	^23^Cs_2_MoCl_6_	180	1000	26	473
	^24^CsPbCl_3_:*x*%Yb^3+^/Er^3+^	240	1540	4	360
Metal oxides, nitrides, or fluorides	^2^Na_3–2*x*_Sc_2_(PO_4_)_3_:*x*Eu^2+^	1300	453	74	473
	^25^MgAl_2_O_4_:Mn^2+^/Eu^2+^	1200	516		423
	^26^K_2_SiF_6_:Mn^4+^	25	630	90	448
	^27^Lu_2_SrAl_4_SiO_12_:Cr^3+^	1500	710	77	573

a *T*_c_ is defined as the temperature at which the PL intensity is equal to 100% of the initial PL intensity at 300 K. *T*_c_ can be used as one of the parameters characterizing the anti-TQ properties of a material.

## Mechanism Discussion for Anti-TQ Metal Halides

The design principles of anti-TQ phosphors follow two main avenues: (1) Minimizing the emission losses caused by thermally induced transitions to the crossover point of ground/excited state; (2) Introducing external energy to compensate the emission losses of the emission center. Based on these principles, four alternative mechanisms have been proposed to explain the anti-TQ behavior in metal halides. The first one is the creation of an extra energy level in the band structure (Figure 2a). This energy level can be in the form of a defect (either from the host or from another dopant). At high temperature, energy transfer from this extra energy level to the emission center is activated, providing additional carriers to the emission center, compensating the nonradiative losses.^2,11^ This compensation mechanism is adopted by most Mn doped,^21^ heterovalent atom doped^28^ and multielement-doped^16^ metal halides.

**Figure 2:**
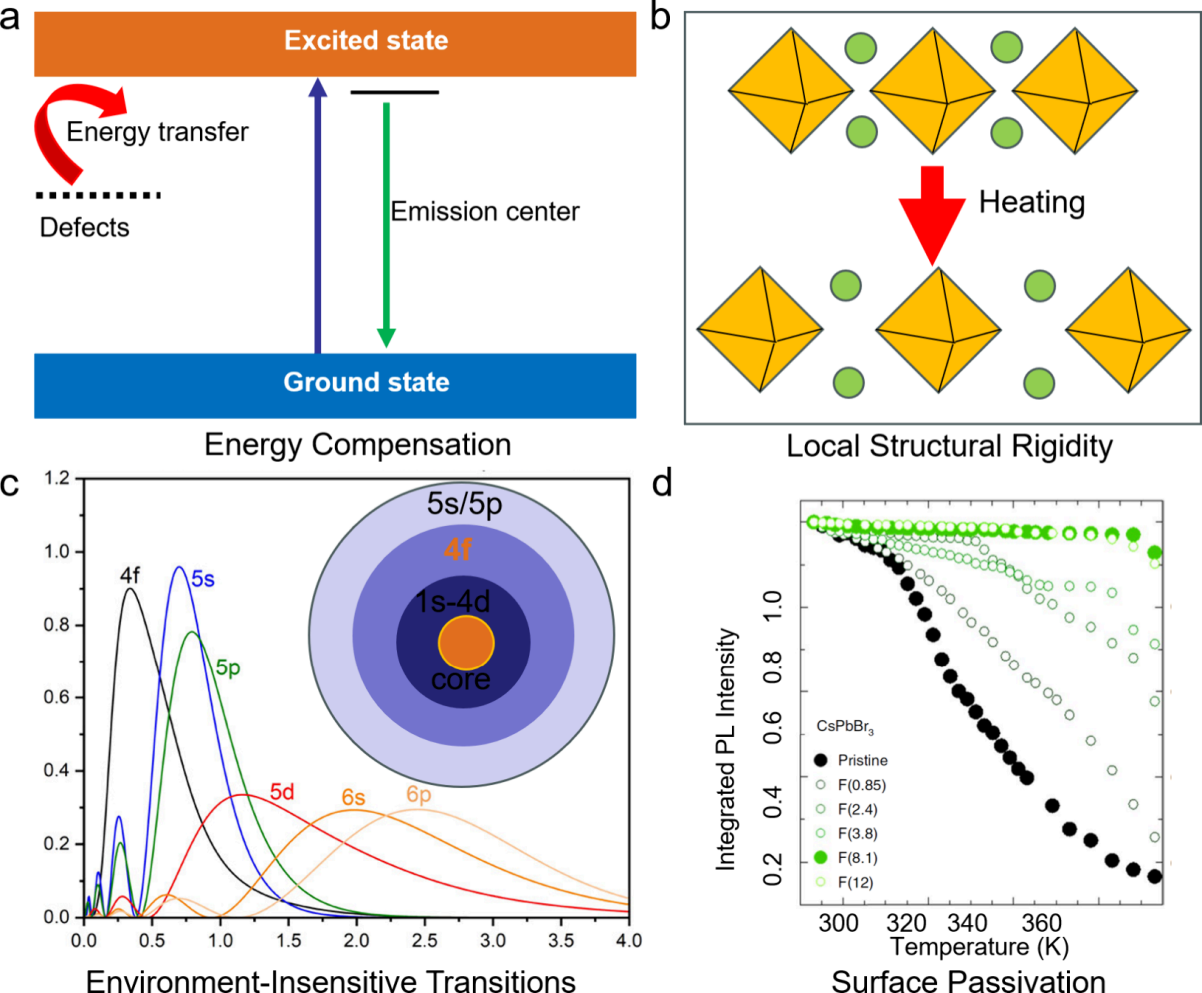
A summary of anti-thermal quenching (anti-TQ) mechanisms in some emissive metal halides. (a) Anti-TQ arising from thermally activated energy transfer from defect (either from the host or from another dopant) to emission center, compensating the emission losse at high temperature. (b) Anti-TQ due to local structural rigidity of 0D metal halides, preventing major changes in the emission center during the lattice expansion at high temperature. (c) Anti-TQ arising from environment-insensitive 4f–4f transitions, as f orbitals are very contracted (the radial distribution function of 4f states is peaked much closer to the nucleus compared to 5s, 5p, and 6s states), therefore they are not engaged in bonding with neighboring orbitals.^37,38^ Reproduced (Adapted) with permission from ref 37. Copyright 2023 American Chemical Society. (d) TQ resistance arising from surface passivation of lead halides perovskite nanocrystals that suppresses the thermally activated nonradiative processes.^17^ Reproduced (Adapted) with permission from ref 17. Copyright 2021, Springer Nature.

Also, if the lattice exhibits high structural rigidity, that it, if the lattice expansion is minimal or even negative at high temperatures, both structure and emission intensity of the emission center will be preserved and the material will exhibit anti-TQ behavior. This high lattice rigidity is common among anti-TQ metal oxide/nitride phosphors.^29,30^ For example, some of A_2_M_3_O_12_ type anti-TQ phosphors exhibit zero-thermal expansion or even negative thermal expansion up to 500 K.^8,31^ However, most metal halides do not have comparable structural rigidity (they have a so-called soft lattice), due to their low lattice formation energy.^32^ Instead, zero-dimensional (0D) metal halides, with their structure characterized by isolated polyhedra, appear to have a local structural rigidity: at high temperature, although these 0D metal halides undergo significant thermal expansion globally, this is mainly accounted for by the elongation of the interpolyhedral distances, while the individual polyhedra (which are also the emission centers) are only marginally expanded (Figure 2b).^4^ Thanks to this local structural rigidity, 0D metal halides have been found to exhibit better anti-TQ performance than their 3D counterparts, as reported in Table 2.^4,33^

**Table 2:** Composition, Dimensionality, Emission Peak (nm), PLQY (%), and *T*_c_ (K) for 0D Metal Halides and Their 3D Counterparts^4,33^

Materials class	Compositions	Dimensionality	Emission peak (nm)	PLQY (%)	*T*_c_ (K)
0D and 3D Sb doped^4^	Rb_3_InCl_6_:*x*%Sb^3+^	0D	521	90	500
	Cs_2_AgInCl_6_:*x*%Sb^3+^	3D	650	51	<300
0D, 1D and 3D Ln halide^33^	C_72_N_12_[Eu_2_I_9_]_2_Eu_4_I_15_	0D	450	95	433
	C_16_N_4_Eu_8_I_24_	1D	450	90	353
	CsEuCl_3_	3D	405	85	313

In the cases of Ln elements, their 4f–4f transitions are essentially environment insensitive, as f orbitals, being very contracted, do not engage in bonding with the orbitals of neighboring atoms (Figure 2c). Most Ln ion-doped metal halides show some degree of anti-TQ.^16,33^ Surface treatment is also an emerging strategy to improve the optical properties of lead halide perovskite nanocrystals,^17,34–36^ and recent findings have revealed how a good surface passivation can efficiently suppress nonradiative processes even at high temperatures. For example, by post-treatment with dodecyl dimethylammonium fluoride (DDAF), CsPbBr_3_ nanocrystals at 373 K preserves 90% of their room temperature PL (Figure 2d).^17^

In the following sections, we will divide the metal halides into three types: (1) metal halides doped with various metal ions (for example Mn^2+^, Sb^3+^, Mo^4+^). We start with this class of materials as historically they were the first ones to be investigated; (2) metal halides doped with Ln ions; (3) surface treated lead halides nanocrystals, and discuss their anti-TQ behavior case by case.

## Anti-TQ Metal Halides with Metal Ion Doping

### Mn^2+^ Doped Cases

Mn^2+^ doping was the first reported strategy to achieve anti-TQ metal halide phosphors. As anticipated in the introduction, in 2017 Yuan et al. synthesized Mn^2+^ doped CsPbCl_3_ nanocrystals and investigated their temperature dependent PL behavior. The PL intensity of Mn^2+^ increased with heating, an effect that was attributed to the accelerated energy transfer from the host to Mn^2+^ dopant states, indicating a mild anti-TQ behavior from 60 to 300 K.^14^ In another work, Pinchetti et al. observed that, in Mn^2+^ doped CsPbCl_3_ nanocrystals, the Mn^2+^ emission exhibits anti-TQ behavior in the 60–300 K range and TQ behavior in the 3.5–60 K range, see Figure 3a.^39^ This anti-TQ behavior was explained by a two-step sensitization pathway: energy transfer from excitonic states to trap states and then to Mn^2+^ dopant states (Figure 3b). These initial temperature dependent PL studies in the low temperature range uncovered the potential for Mn^2+^ doped CsPbX_3_ as anti-TQ phosphors.

**Figure 3:**
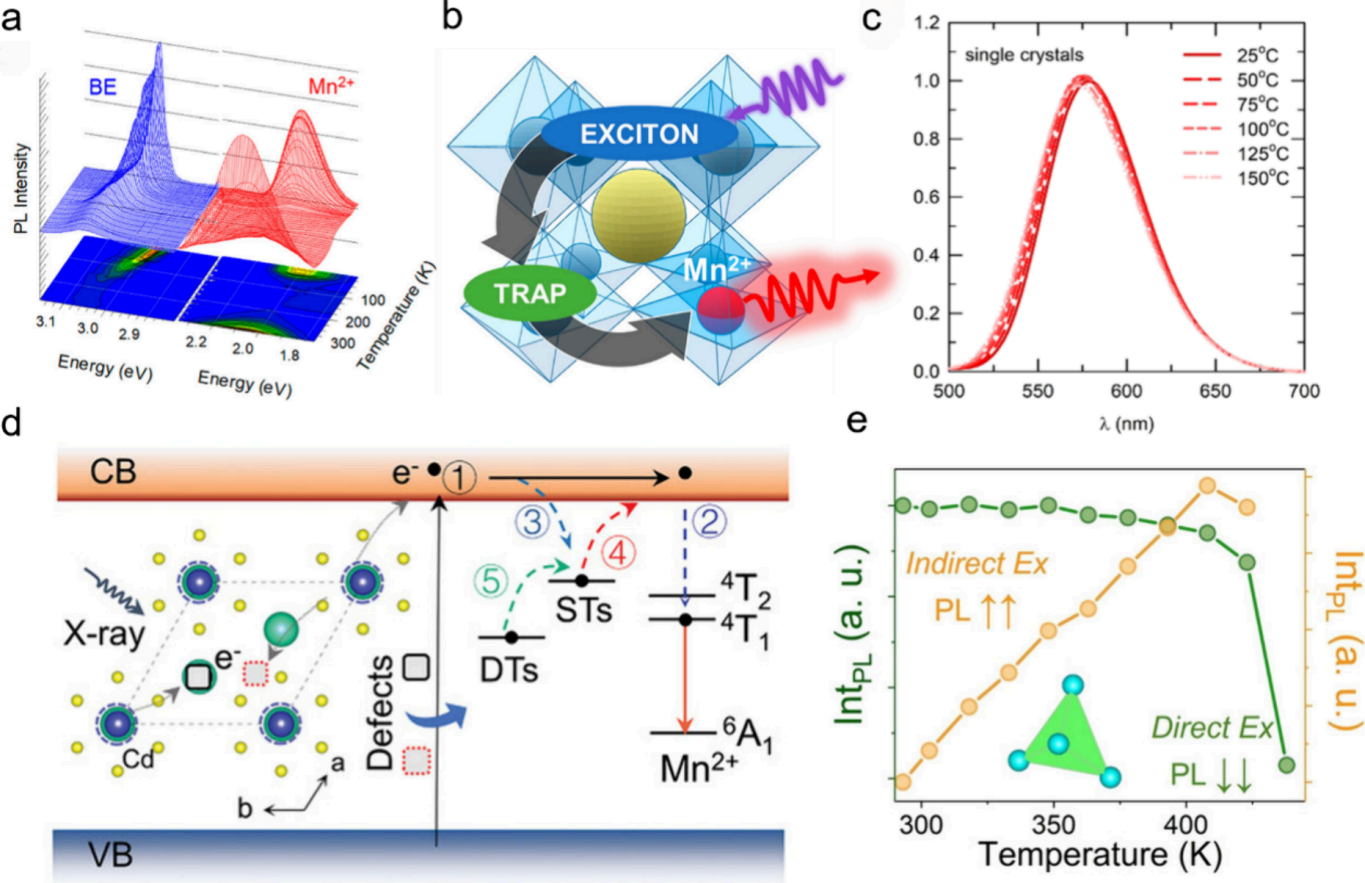
Mn^2+^ based anti-TQ metal halides. (a) Temperature dependent dual-emission of Mn^2+^ doped CsPbCl_3_ nanocrystals at 3.5–300 K and (b) the proposed two-step sensitization model.^39^ Reproduced (Adapted) with permission from ref 39. Copyright 2019 American Chemical Society. (c) Temperature dependent PL spectra of Rb_3_(Cd_1–*x*_Mn_*x*_)_2_Cl_7_ microcrystals at 25–150 °C.^21^ Reproduced (Adapted) with permission from ref 21. Copyright 2022 American Chemical Society. (d) A simplified model to describe the zero-TQ processes of the CsCdCl_3_:5%Mn^2+^,0.1%Zr^4+^ powders, including the formation of excitons (①), radioluminescence (①②), trapping of electrons (③), detrapping of electrons/thermal ionization (④) and energy transfer (⑤).^43^ Reproduced (Adapted) with permission from ref 43. Copyright 2023 John Wiley and Sons. (e) Excitation-dependent anti-TQ behavior of (Gua)_2_MnBr_4_ single crystals.^44^ Under 360 nm excitation, the phosphor shows TQ behavior. Conversely, when excited at 405 nm, it shows anti-TQ behavior. Reproduced (Adapted) with permission from ref 44. Copyright 2025 John Wiley and Sons.

In 2018, Ji et al. investigated the temperature dependent PL of Mn^2+^ doped CsPbCl_3_ nanocrystals with sizes from 5.0 to 17 nm in a 280–400 K temperature range.^40^ They demonstrated that the anti-TQ behavior is size dependent. The maximum PL intensities from Mn^2+^ states were observed at 260, 260, 280, 300, and 340 K for nanocrystals with sizes of 5.0, 7.2, 8.7, 13, and 17 nm, respectively. The authors claimed that the reduced surface trap density in larger nanocrystals might contribute to the suppression of nonradiative processes at high temperatures. In 2022, Kim et al. reported the synthesis of Rb_3_(Cd_1–*x*_Mn_*x*_)_2_Cl_7_ microcrystals exhibiting zero-TQ up to 150 °C.^21^ As shown in Figure 3c, the PL intensity was not quenched and was only slightly blue-shifted at increasing temperature, from 25 to 150 °C. This anti-TQ property was attributed to thermally assisted energy transfer from defect states to luminescent centers. The activation energy of such defects states was estimated to be around 0.7–0.8 eV by thermoluminescence (TL) measurements, based on the formula *E*_a_ (eV) *= T*(K)/500, where *E*_a_ represents the activation energy and *T* is the peak position in the TL spectra.^41^ In 2023, Liu et al. prepared Mn^2+^ doped Cs_2_CdCl_4_ crystals with Ruddlesden–Popper phase.^42^ This phosphor had a long-persistent luminescence with decay lifetimes ranging from 450 to 600 s, and exhibited anti-TQ PL in the 300–500 K range. In the same year, Zhou et al. prepared Mn^2+^/Zr^4+^ co-doped CsCdCl_3_ crystals featuring zero-TQ radioluminescence up to 448 K under X-ray excitation. In that system, doping with heterovalent ions (Zr^4+^) introduces structural defects in the lattice, which can then transfer energy to the Mn^2+^ dopants at elevated temperature (Figure 3d).^43^ In 2024, Wu et al. developed a hybrid organic–inorganic (Gua)_2_MnBr_4_ (Gua = *N*,*N*′-diphenylguanidinium) single crystal that also exhibited anti-TQ behavior up to 400 K under X-ray excitation.^44^ Such anti-TQ behavior was found to be excitation dependent: the anti-TQ was observed when the samples were excited by 405 nm light (indirect excitation), while TQ was observed when excited by 365 nm light (direct excitation), as shown in Figure 3e. Through molecular dynamic simulations, the authors claimed that the Mn-ligands distance increases with increasing temperature, while the Mn–Mn distance in the lattice surprisingly decreases at increasing temperature. This abnormal shortening of the Mn–Mn distance was believed to contribute to the anti-TQ photoluminescence behavior of the (Gua)_2_MnBr_4_ crystals.

### Sb^3+^ Doped Cases

Sb^3+^ is another widely used dopant in anti-TQ metal halide phosphors. Mn^2+^ halides usually exhibit fixed orange-red emission (when six-coordinated) or green emission (when four-coordinated), while emission from Sb^3+^ halides can be tuned from the visible range to the NIR by adjusting its coordination environment.^45^ In 2022, Li et al. reported the synthesis of Sb^3+^-doped (BTPP)_2_MnCl_4_ (BTPP = Benzyl triphenyl phosphonium) single crystals exhibiting dual emission: self-trapped exciton (STE) emission from [SbCl_4_]^−^ and an emission due to the ^4^T_1_-^6^A_1_ transition from Mn^2+^ states. The STE emission preserved 72.5% of its 298 K PL intensity at 420 K (Figure 4a). Such TQ resistance was attributed to the efficient energy transfer from the [MnCl_4_]^2–^ host to [SbCl_4_]^−^, promoting STE emission at elevated temperatures.^46^ In 2024, Zhang et al. investigated the temperature dependent PL spectra of Sb^3+^ doped Cs_2_ZnCl_4_ crystals in the 80–480 K range. Green emission (550–565 nm) was ascribed to the [ZnCl_4_]^2–^ host (STE-2), while NIR emission (690–780 nm) originated from the [SbCl_4_]^−^ dopant (STE-3). As the temperature increased, the STE-2 emission gradually vanished while the STE-3 emission intensified between 80 and 220 K and slightly decreased between 220 and 380 K, indicating a mild anti-TQ behavior (Figure 4b). The authors proposed that the lattice softening at high temperature promotes the formation of STEs, thereby enhancing the luminescence intensity.^28^

**Figure 4:**
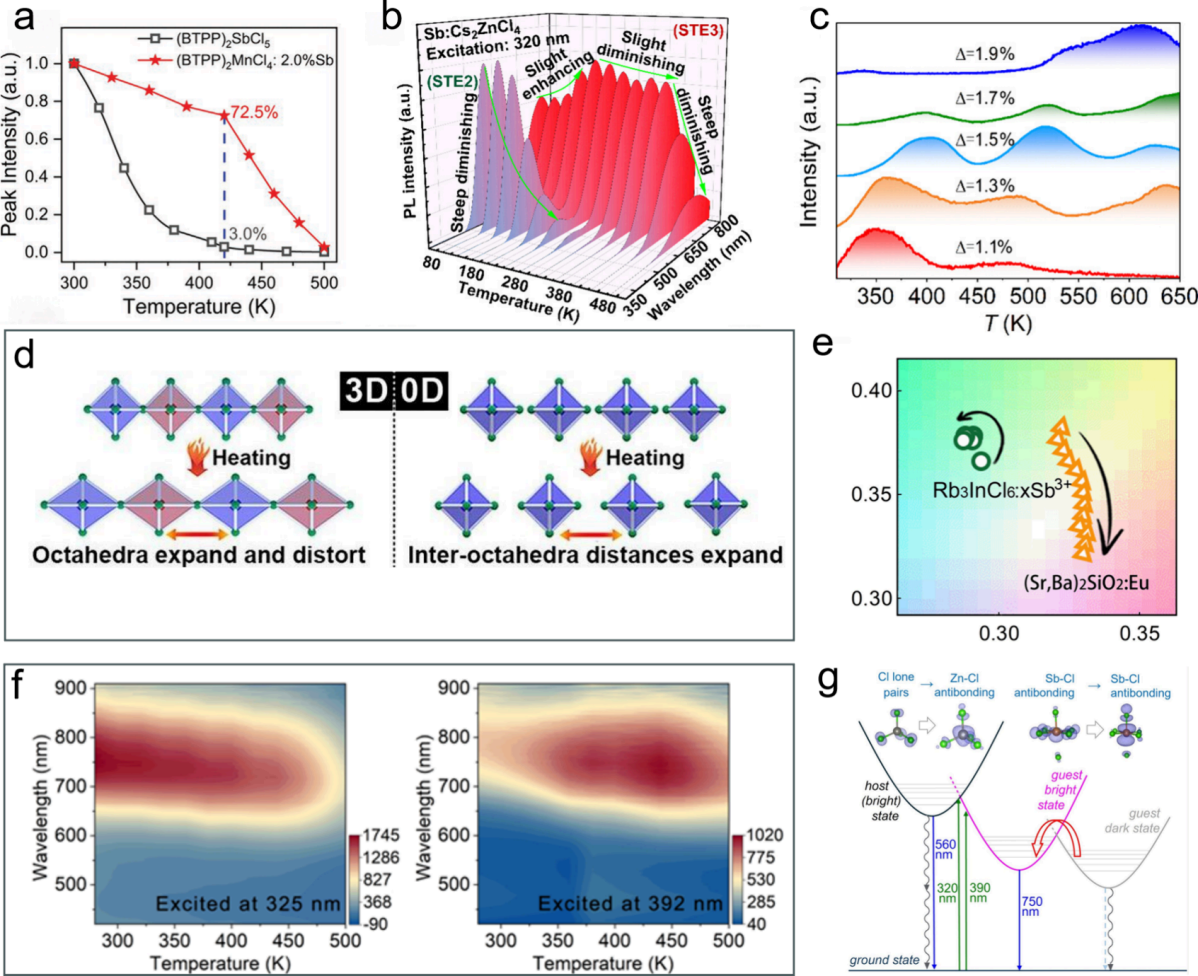
Sb^3+^ based anti-TQ metal halides. (a) Temperature dependent PL intensity of (BTPP)_2_SbCl_5_ and (BTPP)_2_SbCl_5_:2.0%Sb^3+^ in the 298–500 K range.^46^ Reproduced (Adapted) with permission from ref 46. Copyright 2022 John Wiley and Sons. (b) PL spectra of the dual emissive Sb^3+^:Cs_2_ZnCl_4_ crystals in the 80–480 K range.^28^ Reproduced (Adapted) with permission from ref 28. Copyright 2024 American Chemical Society. (c) Thermoluminescence spectra of Rb_3_InCl_6_:*x*Sb^3+^ with different deficient values (Δ) of InCl_3_ precursors during synthesis. (d) Proposed thermal-lattice expansion model for 0D and 3D metal halide frameworks. (e) CIE chromaticity coordinates of the Rb_3_InCl_6_:*x*Sb^3+^ prototype white light-emitting diodes compared to the commercial green rare-earth oxide phosphor-based white light-emitting diodes, under increasing current (from 50 to 2000 mA).^4^ Reproduced (Adapted) with permission from ref 4. Copyright 2024 American Chemical Society. (f) Temperature dependent PL intensities of Cs_2_ZnCl_4_:0.29%Sb^3+^ single crystals at two different excitation wavelengths (left) 325 nm and (right) 392 nm. (g) Proposed model for electronic excitations in Cs_2_ZnCl_4_:Sb^3+^. The green, blue, and curly gray arrows represent, respectively, absorption, radiative recombination, and nonradiative recombination paths. The broad red arrow refers to the thermally assisted energy transfer from the dark to the bright state.^22^ Reproduced (Adapted) with permission from ref 22. Copyright 2025 John Wiley and Sons.

In 2024, Zhang et al. (our groups) reported Rb_3_InCl_6_:*x*Sb^3+^, in the form of powders, as a robust anti-TQ phosphor exhibiting zero-TQ emission up to 500 K^4^. This property appears to stem from the combination of a defect compensation effect and an intrinsic structural rigidity of the isolated octahedra in the 0D structural framework of this material. First, the creation of intentional InCl_3_ deficiencies during the synthesis generated a high density of lattice defects. Defect energy depths of 0.7–1.2 eV were estimated by thermoluminescence measurements (Figure 4c). At increasing temperatures, the defects release energy to the [SbCl_6_]^3–^ centers and thus compensate the nonradiative losses. Additionally, while 0D halides undergo significant thermal expansion, XRD analysis revealed that this is mainly accounted for by the elongation of the interoctahedral distances, while no substantial distortion of the [SbCl_6_]^3–^ centers was observed. Thus, the intrinsic rigidity of the emitting sites was considered as the origin of the suppression of nonradiative processes at high temperatures (Figure 4d). Using this anti-TQ metal halide, high-power white light-emitting diodes were fabricated. These devices maintained stable PL intensity and chromaticity under currents in the 50–2000 mA range (Figure 4e), with performances comparable to those of commercial metal oxide/nitride phosphors.^2,47^

Later on, a work from our groups reported an anti-TQ NIR metal halide phosphor, Cs_2_ZnCl_4_:xSb^3+^, in the form of centimeter-scale crystals.^22^ The sample had an PL emission peaked at 745 nm, with a photoluminescence quantum yield (PLQY) of up to 75%, and exhibited robust anti-TQ behavior up to 500 K. The anti-TQ behavior of this sample was excitation-dependent, appearing at wavelengths above 370 nm (Figure 4f). Atomistic simulations suggested that this anti-TQ behavior stems from the interplay among host/dopant, dark/bright excited states, phonons, and thermal energy. As shown in Figure 4g, the high-energy photons (e.g., 320 nm, green upward arrow) excite mainly the host, while the low-energy photons (e.g., 390 nm, green upward arrow) directly excite the dopant. Direct guest excitation leads to a higher population of the guest bright state (purple curved line). In this scenario, the limited number of dopant states quickly become saturated, forcing excess carriers into the dark state. At high temperature, the thermally accelerated transition from dark state to bright state compensates the nonradiative loss of the bright state (broad red arrow), a process that should rationalize the robust anti-TQ emission. Instead, when the host is excited, the guest state population is lower, as the host–guest transition is thermally activated. Thus, the carriers recombine before they can overcome the significant energy barrier to the dark state. As a result, the energy transfer from dark state to bright state is not feasible in those excitation conditions. These anti-TQ NIR phosphors were then exploited to fabricate a LED that exhibited stable emission intensity up to 1000 mA, and its high penetration depth was demonstrated on real-life objects.

### Mo^4+^/W^4+^ Alloy/Doping Based Cases

In 2022, Liu et al. synthesized Cs_2_MoCl_6_ and Cs_2_WCl_6_ single crystals having NIR emission with a PLQY of 26%.^23^ As shown in Figure 5a, the NIR emission intensity of the Cs_2_MoCl_6_ crystals gradually decreased when the temperature increased from −180 to 60 °C, but subsequently increased from 60 to 200 °C, hence demonstrating robust anti-TQ behavior (Figure 5a). Luminescence quenching from −180 to 60 °C was attributed to phonon assisted nonradiative decay, while anti-TQ at high temperature was ascribed to both thermally facilitated STE formation and thermally activated energy transfer from trapped charge carriers to STEs. In 2024, Li et al. synthesized Mo^4+^ doped Cs_2_ZrCl_6_ powders with a broadband NIR emission centered at 970 nm, originating from transitions between the lowest ^1^T_2_ state and a manifold of ^3^T_1_ states.^48^ This NIR phosphor showed mild TQ resistance, maintaining 66.9% of its room temperature PL intensity at 150 °C (Figure 5b), a performance that is comparable to that of most commonly reported Cr^3+^-doped metal oxide phosphors with similar NIR emission.^49^ Shubham et al. later prepared alloyed Cs_2_Zr_1–*x*_Mo_*x*_Cl_6_ powders exhibiting dual emission, from [ZrCl_6_]^2–^ STE and from [MoCl_6_]^2–^ d-d transitions. Notably, the [MoCl_6_]^2–^ NIR emission increased as the temperature increased from 250 to 300 K, thus showing anti-TQ behavior (Figure 5c). The authors proposed that thermal heating softens the lattice, facilitating vibronically coupled d–d transitions and releasing trapped charge carriers.^50^

**Figure 5:**
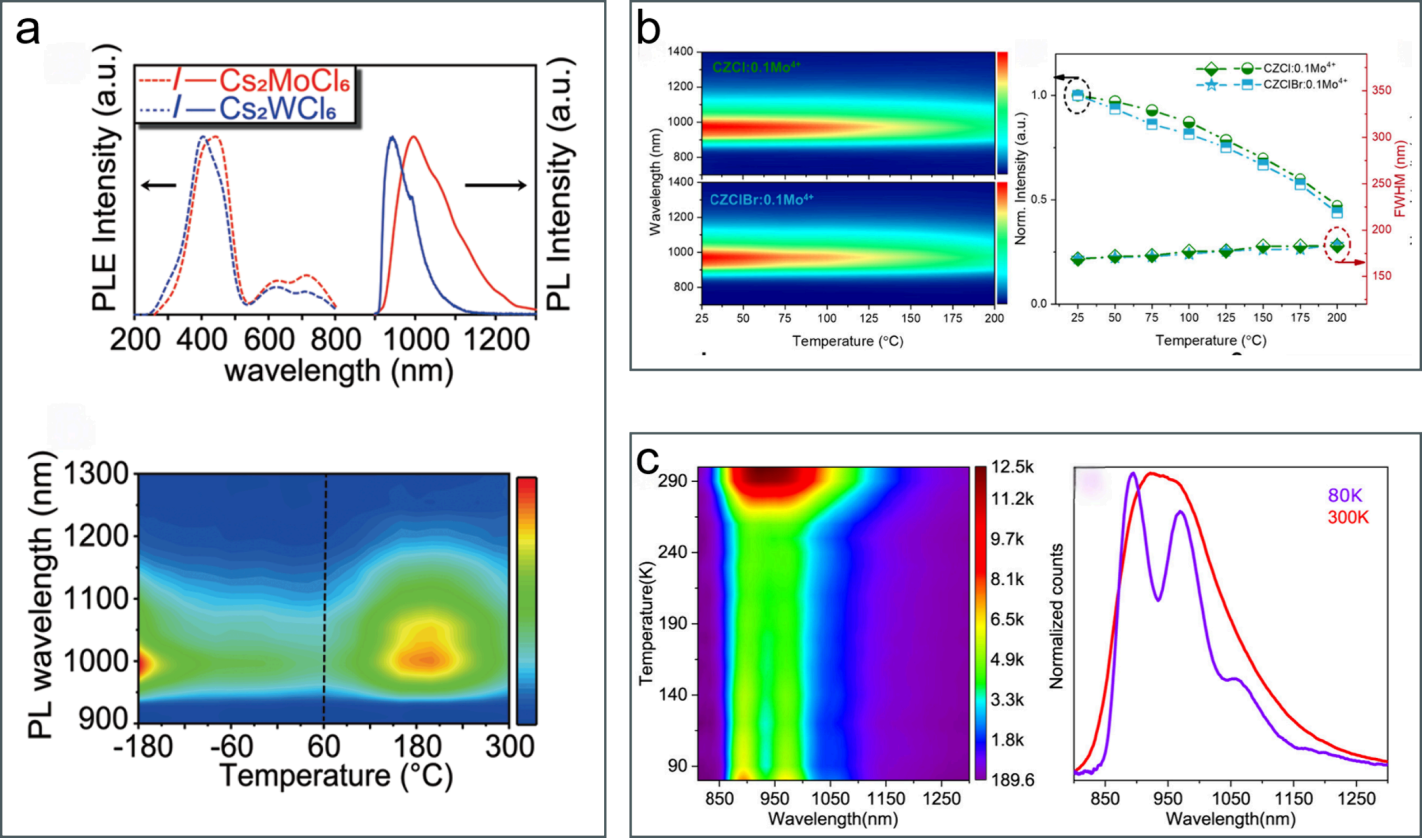
Mo^4+^/W^4+^ based anti-TQ metal halides. (a) PL, PLE and temperature dependent PL spectra of Cs_2_MoCl_6_ single crystals.^23^ Reproduced (Adapted) with permission from ref 23. Copyright 2022 John Wiley and Sons. (b) Temperature dependent PL spectra of Cs_2_ZrCl_6_:0.1Mo^4+^ and Cs_2_ZrCl_6–*x*_Br_*x*_:0.1Mo^4+^ powders, and their temperature dependent PL intensity and full width at half-maximum (FWHM).^48^ Reproduced (Adapted) with permission from ref 48. Copyright 2024 American Chemical Society. (c) Temperature dependent alloyed Cs_2_Zr_1–*x*_Mo_*x*_Cl_6_ powders and its normalized PL spectra at 80 and 300 K, respectively.^50^ Reproduced (Adapted) with permission from ref 50. Copyright 2024 American Chemical Society.

### Hybrid Organic–Inorganic Anti-TQ Metal Halides

Some hybrid organic–inorganic metal halides also show anti-TQ behavior through thermal activated energy transfer from metal halides to organic emitters.^51^ For example, (Ph_4_P)_2_Cd_2_Br_6_ maintains stable emission intensity across a wide temperature range from 100 to 320 K (*T*_c_ = ∼50 °C).^51^ As this energy transfer process usually involves a singlet–triplet transition, these phosphors also show persistent phosphorescence.^20,51,52^

## Anti-TQ Metal Halides with Ln Ion Doping

Yu et al. incorporated Yb^3+^, Er^3+^, and Ho^3+^ into CsMnCl_3_ single crystals with trigonal structure (space group *R*3̅*m*) and achieved anti-TQ emission above room temperature.^53^ In temperature dependent photoluminescence (PL) experiments, the Mn-related emission decreased significantly with increasing temperature, while the Ln^3+^ emission intensity in the near-infrared (NIR) region exhibited zero TQ up to 400 K. As reported in Figure 6a, the [MnCl_6_]^4–^ host absorbs UV–visible light and undergoes transitions from ^6^A_1g_(S) states to various excited states (blue upward arrow). After that, relaxation can occur through three pathways: (1) radiative transition to the ground state, with emission of red PL (^4^T_1g_(G)→^6^A_1g_(S), purple downward arrow); (2) crossover to the ground/excited-state cross point followed by nonradiative transitions (TQ, dashed arrow); (3) overcoming the energy barrier between the host and dopants, followed by energy transfer to Ln ions (energy transfer, curved red arrow), activating their NIR emission. High temperatures result in TQ by providing sufficient energy to reach the crossover point, thus reducing emission intensity from [MnCl_6_]^4–^ states. At the same time, higher temperatures accelerate energy transfer from [MnCl_6_]^4–^ to Ln^3+^ dopants, which explains the anti-TQ behavior of the NIR emission.

**Figure 6:**
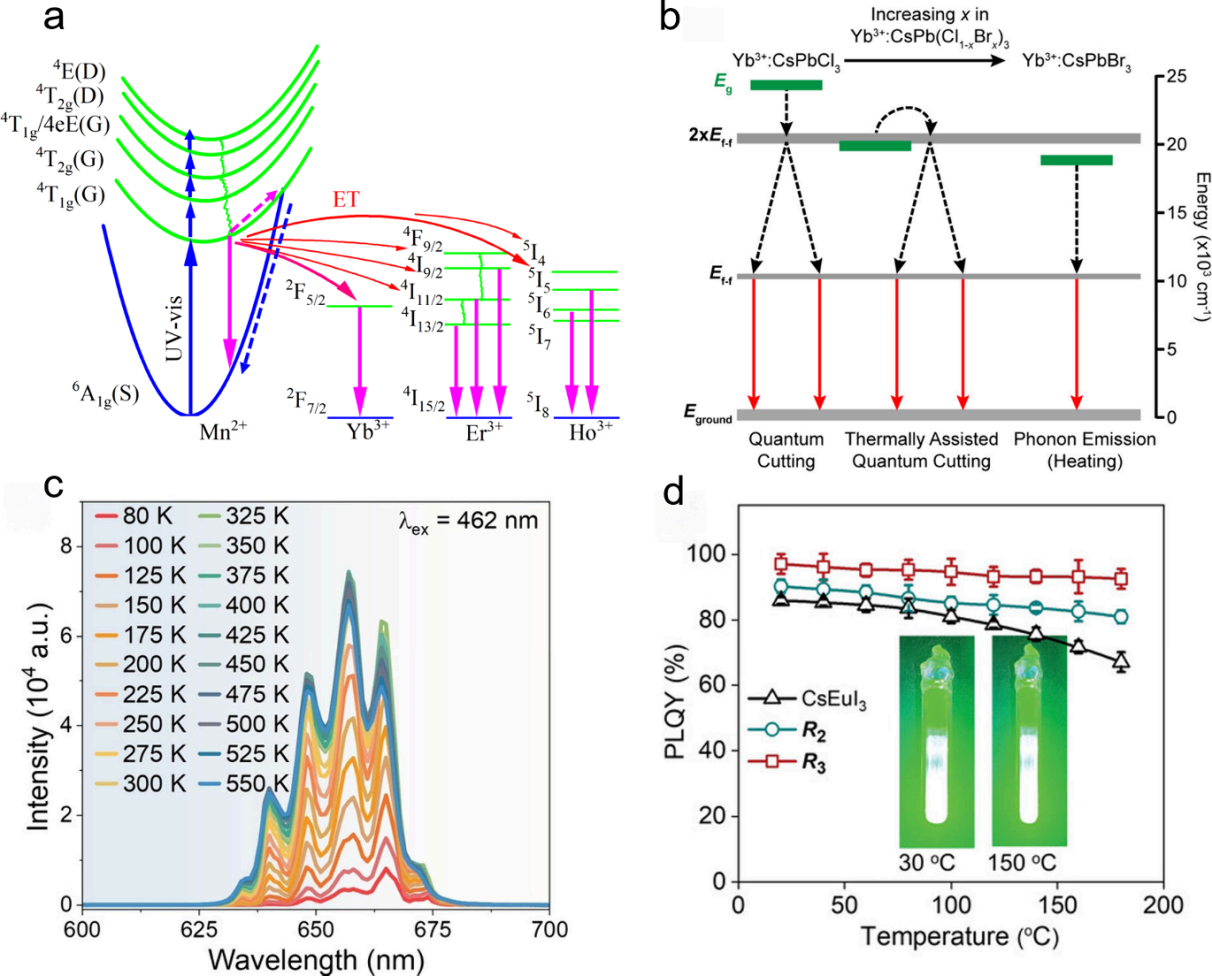
Ln based anti-TQ metal halide phosphors. (a) Energy level diagram and electron dynamics of CsMnCl_3_:Ln^3+^ (Ln = Yb, Er, and Ho) single crystals.^53^ Reproduced (Adapted) with permission from ref 53. Copyright 2022. Elsevier. (b) Quantum-cutting energy transfer in Yb^3+^:CsPb­(Cl_1–*x*_Br_*x*_)_3_ nanocrystals. The quantum-cutting process is thermally activated.^15^ Reproduced (Adapted) with permission from ref 15. Copyright 2023 American Chemical Society. (c) Temperature dependent PL spectra of Sb^3+^/Yb^3+^ co-doped Cs_2_NaHoCl_6_ single crystals in the temperature range from 80 to 550 K.^16^ Reproduced (Adapted) with permission from ref 16. Copyright 2023 John Wiley and Sons. (d) Temperature-dependent PLQY of 3D (triangle), 1D (circle) and 0D (square) Eu halides powders in the 25–180 °C temperature range.^33^ Reproduced (Adapted) with permission from ref 33. Copyright 2024 John Wiley and Sons.

Zhao et al. and Roh et al. reported anti-TQ Yb^3+^-doped CsPbX_3_ nanocrystals in 2023.^15,54^ Zhao et al. observed a 2.5-fold enhancement of the Er^3+^ luminescence (peak at 1540 nm) from 298 to 356 K in Yb^3+^/Er^3+^ co-doped CsPbCl_3_ nanocrystals,^54^ while Roh et al. found a >100 times enhanced Yb^3+^ luminescence intensity from 5K to room temperature in Yb^3+^ doped CsPb­(Cl_*x*_Br_1–*x*_)_3_ nanocrystals. Both groups proposed a thermally assisted quantum cutting process to explain the observed anti-TQ effect.^15^ As shown in the left side of Figure 6b, Yb^3+^:CsPbCl_3_ has E_g_ > 2 × E_f–f_ and is capable of quantum cutting at all temperatures, with release of excess energy via phonon emission. However, Yb^3+^:CsPbCl_3–*x*_Br_*x*_ (0.5≤ *x* ≤ 1.00) has E_g_ ≈ 2 × E_f–f_, so that its lowest donor transition is slightly below the lowest Yb^3+^–Yb^3+^ transition (middle part of Figure 6b). At high temperatures, thermal band gap widening and line broadening assist these transitions from donor to Yb^3+^-Yb^3+^ and thus improve the quantum cutting process. In contrast, Yb^3+^:CsPbBr_3_ has E_g_ < 2 × E_f–f_, so it does not allow a quantum cutting process (right side of Figure 6b). Additionally, heating was also believed to remove excess water molecules from the surface of the nanocrystals and this process was hypothesized to heal the surface traps and further suppress the TQ of Yb^3+^/Er^3+^ co-doped CsPbCl_3_ nanocrystals.^54^

Wang et al. prepared Sb^3+^/Yb^3+^ co-doped Cs_2_NaHoCl_6_ single crystals in which the Ho^3+^ luminescence exhibited anti-TQ in the 80–500 K range (Figure 6c).^16^ They attributed anti-TQ to environment-inert 4f–4f transitions. Additionally, they claimed that thermal expansion of the matrix reduced the local concentration of Ho^3+^ ions and diminished the concentration quenching effect caused by nonradiative energy transfer between neighbor dopants. However, this lattice-expansion induced concentration reduction should be minor. For example, based on the temperature dependent XRD, the volume of Rb_3_InCl_6_:xSb^3+^ lattice expands only ∼3.7% and thus will only result ∼3.7% concentration reduction of Sb^3+^ dopants from 300 to 500 K.^4^

Some Ln ions (Eu^2+^, Ce^3+^) undergo 4f-5d transitions whose PL emission is more environment-sensitive compared to the more standard Ln^3+^ case, where only 4f–4f transitions can occur. In 2024, Han et al. developed a series of hybrid organic–inorganic Eu­(II)-based halides powders with structural dimensionality ranging from 0D to 1D and 3D.^33^ The 0D Eu halide exhibited optimal anti-TQ, with PL decreasing only slightly from 98% to 93% in the 25–180 °C temperature range (Figure 6d). These Eu halides feature a narrower emission (FWHM ≈43 nm) compared to commercial metal nitride phosphors (β-SiAlON: Eu^2+^, FWHM ≈54 nm) and their anti-TQ properties are better than those of most lead-based halide perovskites.

## Anti-TQ in Lead Halide Perovskites Following Surface Treatments

Lead halide perovskites have been extensively applied in electroluminescence devices, although they suffer from rapid efficiency roll-off at high operating temperatures. To address this, Liu et al. treated CsPbBr_3_ nanocrystals postsynthesis with dodecyl dimethylammonium fluoride (DDAF, Figure 7a).^17^ The DDAF-treated CsPbBr_3_ nanocrystals exhibited only slight emission quenching (10% intensity loss) from 298 to 373 K, compared to nearly complete quenching in pristine nanocrystals (Figure 7a). The authors proposed that the fluorine-rich surface leads to a wide-bandgap type-I ‘core–shell’ structure, suppressing carrier trapping while improving thermal stability and charge injection. These nanocrystals were incorporated into light-emitting diodes that achieved 19.3% external quantum efficiency (EQE) at 350 cd·m^–2^ and retained nearly 80% of their room-temperature EQE at 343 K. They subsequently tested various anions (OH^–^, SO_4_^2–^, F^–^) for the CsPbBr_3_ surface treatment, and discovered that they all lead to the formation of wide-bandgap passivation layers (PbSO_4_, Pb­(OH)_2_, and PbF_2_). The results indicated that not only F^–^ but also SO_4_^2–^ ions improve the TQ resistance, preserving 70% of the room temperature PL intensity at 373 K.^55^ Wang et al. further incorporated the fluoride treated CsPbBr_3_ nanocrystals into silica matrices. The resulting fluorine-treated CsPbBr_3_/silica composites exhibited a high PLQY of 94.5% and preserved 83% of their room temperature PL intensity at 348 K (Figure 7b), plus a complete PL recovery after a thermal cycling (up to 403 K). Also, they retained 95.6% of their initial PL intensity after 1055 h of intense blue light irradiation (350 mW·cm^–2^).^34^

**Figure 7:**
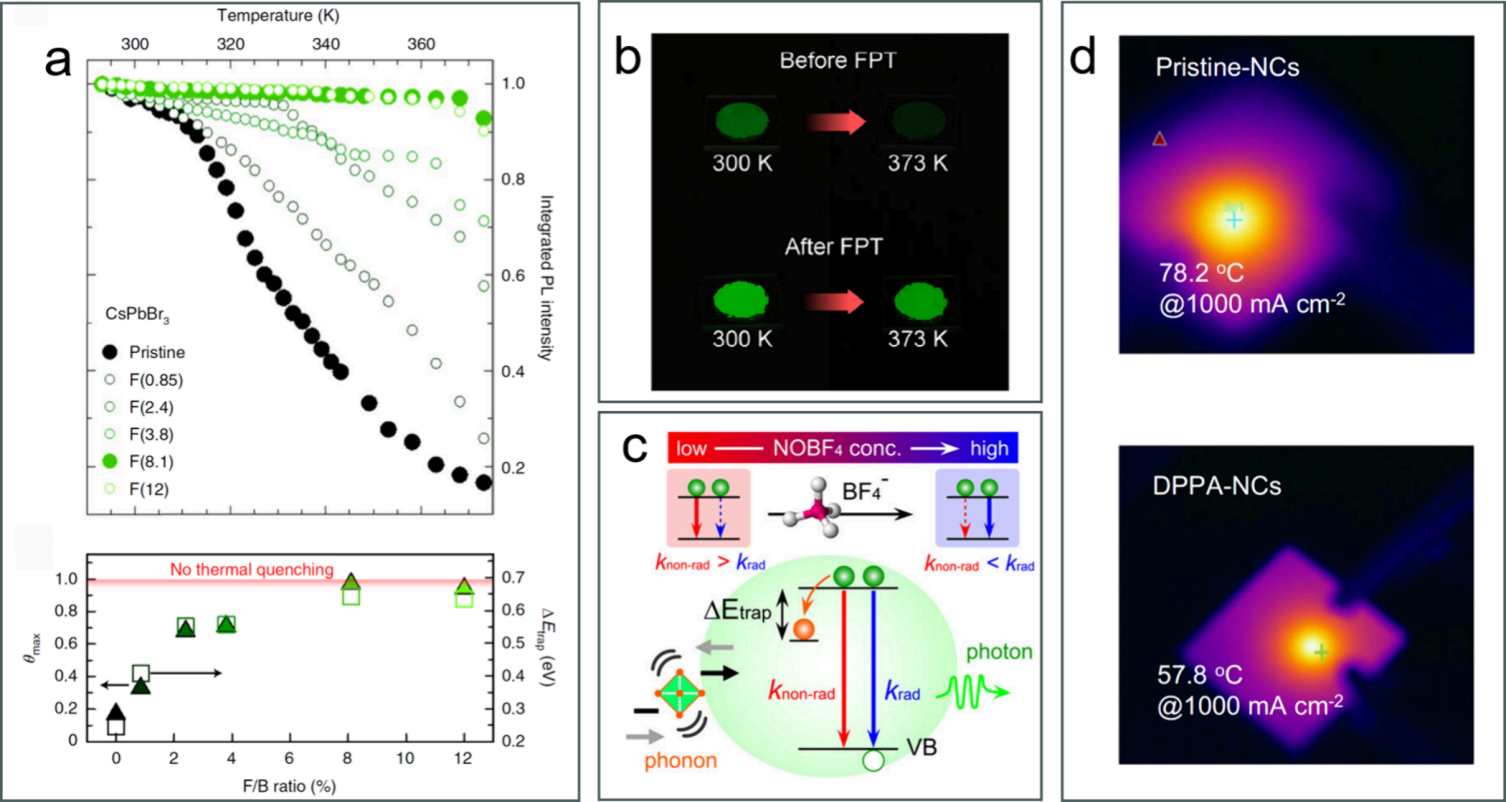
Anti-TQ lead halide perovskite nanocrystals with surface treatment. (a) Integrated PL intensity of pristine and fluoride-treated CsPbBr_3_ nanocrystals at increasing temperatures, from 293 to 373 K, and TQ resistance (θ_max_) and Δ*E*_trap_ of CsPbBr_3_ nanocrystals as a function of the F/Br ratio.^17^ Reproduced (Adapted) with permission from ref 17. Copyright 2021 Springer Nature. (b) Comparison of TQ resistance between the untreated CsPbBr_3_ nanocrystals and fluorine-treated CsPbBr_3_/silica composites.^34^ Reproduced (Adapted) with permission from ref 34. Copyright 2025 John Wiley and Sons. (c) The proposed TQ resistance model of NOBF_4_ treated CsPbBr_3_ nanocrystals.^18^ Reproduced (Adapted) with permission from ref 18. Copyright 2024 American Chemical Society. (d) Comparison of the QLED operation temperature between the pristine nanocrystals and the diphenylphosphoryl azide (DPPA) treated ones.^56^ Reproduced (Adapted) with permission from ref 56. Copyright 2024 Springer Nature.

In 2024, Jeon et al. reported that a NOBF_4_ treatment improves both PLQY and TQ resistance of CsPbBr_3_ nanocrystals. In particular, the treated nanocrystals exhibited almost no thermal quenching in the 80–250 K range, while the untreated sample lost 45% of its PL intensity over the same temperature range (Figure 7c).^18^ Dai et al. introduced diphenylphosphoryl azide (DPPA) during the synthesis of CsPb­(Br/I)_3_ nanocrystals.^56^ These ligands effectively passivated the surface of the nanocrystals, increasing the PLQY to near unity values and reducing Joule heating. The highly thermally conductive DPPA ligands were hypothesized to transfer heat efficiently and thus reduce the operation temperature of the devices. As shown in Figure 7d, at an applied current of 1000 mA·cm^–2^, the pristine-nanocrystals based QLED featured an operating temperature of 78.2 °C, while the operating temperature of the DPPA nanocrystals based QLED was only 57.8 °C. These thermally optimized QLEDs exhibited an ultrabright luminance of 390,000 cd·m^–2^, a peak external quantum efficiency of 25%, and an operational half-life of 20 h.

## Conclusion

In summary, we have provided an overview of a very young field of research, that of anti-TQ metal halide phosphors. This focus review covers a very short time span, starting from 2017 ^14^ until the present (2025). Although significant progress has been made in this relatively short time span, we can definitely state that research in this area is still in its early stages. An important point that we want to stress in this review is that mainstream anti-TQ metal oxide/nitride phosphors are typically synthesized above 1000 °C due to their high lattice formation energies (Table 1). In contrast, softer-lattice metal halides with comparable anti-TQ properties can be synthesized below 200 °C or even at room temperature (Table 1), thus offering potential advantages for metal halides in terms of low-temperature processing and energy efficiency.

We have seen how anti-TQ metal halides can be grouped in three classes: (i) phosphors based on metal ions (Mn^2+^, Sb^3+^, Mo^4+^/W^4+^) or on (ii) Ln ions, and (iii) surface-passivated lead halide nanocrystals. For the Ln-based metal halides, the environment-insensitive 4f–4f transitions of Ln^3+^ ions appear promising for anti-TQ phosphors. However, their fixed emission peaks cannot be adjusted by changing the dopant environment. In principle, ions like Eu^2+^ and Ce^3+^, with their hybrid 4f-5d transitions, show greater potential, offering both anti-TQ behavior and tunable emission colors due to the high sensitivity of the 5d orbitals to coordination environments. However, Eu^2+^ halides are very hydrophobic and easy to be oxidized (in this respect they are even more labile than lead halides), and Ce^3+^ halides only show mild TQ resistance. For what concerns metal halides doped with other metal ions, some of them (e.g., Rb_3_InCl_6_:Sb^3+^, Cs_2_ZnCl_4_:Sb^3+^, Rb_3_Cd_2_Cl_7_:Mn^2+^) exhibit robust zero-thermal quenching up to 500 K and have already been integrated successfully into prototype high-power lighting devices. On a negative side, the anti-TQ mechanisms proposed to date are still unclear and sometimes they contradict each other. For example, some studies suggest that high temperatures distort emission centers and quench STE emission,^4,46^ while others propose that they increase available STE states and enhance emission intensity.^23,28,50^ Also, some of metal halides exhibit excitation dependent anti-TQ behavior,^4,44^ which is rarely reported in metal oxides/nitrides anti-TQ phosphors and may hinder their application in WLED. A thorough mechanistic investigation is needed to reconcile these observations. Additionally, low-dimensional metal halides have wide bandgaps and low conductivity, limiting their use in electroluminescent devices.

For what concerns lead halide nanocrystals, they have been successfully employed in electroluminescent devices with superior thermal stability. However, ideal zero-TQ remains unrealized, and most reported nanocrystals show only mild TQ resistance (*T*_c_ < 298 K). This is understandable since surface treatments only form a thin layer on the surface of the nanocrystal. Also, this ligand treatment has been reported only for CsPbBr_3_ nanocrystals, while the possibility to extend anti-TQ behavior also to CsPbI_3_ and CsPbCl_3_ nanocrystals is still to be investigated.

Overall, once the above challenges for anti-TQ metal halides are addressed (Figure 8), the application potential on hp-WLED, down-converted displays and high-power lasers will be fully released for this interesting class of materials. In this conclusive section, we also want to lay down some broad guidelines to design an ideal anti-TQ metal halide, based on what we have learned so far: (1) a 0D framework is preferable to a 3D one, since the isolated polyhedra would be less distorted during thermal induced lattice expansion; (2) heterovalent doping (e.g., Sb^3+^ doped Cs_2_ZnCl_4_) could efficiently induce structure defects and thus compensate the nonradiative losses through energy transfer from defects to emitter at high temperature; (3) beyond ligand treatments, researchers should develop additional strategies (e.g., doping or alloying) to further enhance and extend anti-TQ properties of lead halides phosphors.

**Figure 8:**
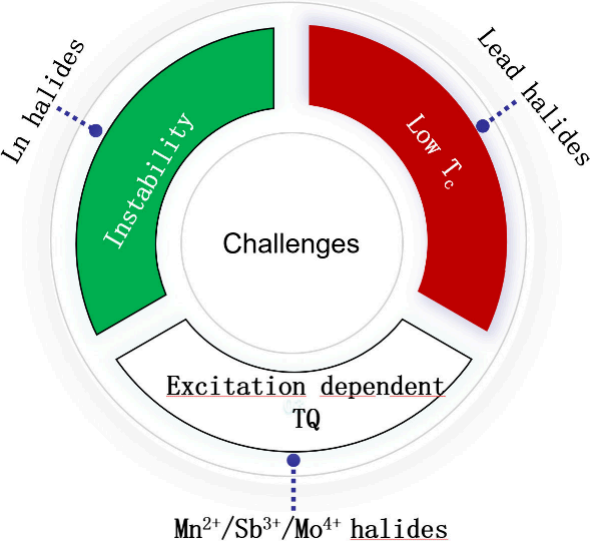
Current challenges for three main classes of anti-TQ metal halides discussed in this review.

Finally, we hope that this focus review will stimulate additional studies toward cost-effective, energy-efficient, and multifunctional anti-TQ metal halide phosphors.
